# The complete chloroplast genome sequence of *Oxystophyllum changjiangense* (Orchidaceae)

**DOI:** 10.1080/23802359.2021.1962766

**Published:** 2021-08-13

**Authors:** Mengting Wang, Jiapeng Yang, Qingyun Xue, Wei Liu, Zhitao Niu, Xiaoyu Ding

**Affiliations:** College of Life Sciences, Nanjing Normal University, Nanjing, China

**Keywords:** *Oxystophyllum changjiangense* (S. J. Cheng & C. Z. Tang) M. A. Clements chloroplast genome, phylogeny, Oxystophyllum

## Abstract

*Oxystophyllum changjiangense* has high economic value due to its wide applications in horticultural and medicinal fields. Here, the first chloroplast genome of *O*. *changjiangense* was sequenced and reported. The chloroplast genome displayed the typical quadripartite structure containing a pair of inverted repeats (IR), a long single-copy region (LSC), and a short single-copy region (SSC). Total 110 genes were found including 76 protein-coding genes, 30 tRNA genes, and four rRNA genes. Phylogeny analysis showed *O*. *changjiangense* has a close relationship with *Phaius* species.

Orchidaceae, one of the largest families in angiosperms, contains approximately 27,000 species distributed in tropical and subtropical regions of the world (Kim et al. 2020). *Oxystophyllum changjiangense* (S. J. Cheng & C. Z. Tang) M. A. Clements 2003 with high ornamental and medicinal value is the only species in *Oxystophyllum*. Chloroplast genomes of angiosperms were wildly used in molecular taxonomy, species evolution, and authentication. Orchidaceae phylogeny was successfully reconstructed using 76 protein-coding genes from the chloroplast genome (Li et al. 2019). Zhu et al. accurately authenticated *Dendrobium officinale* and its five closely related species based on whole chloroplast genomes (Zhu et al. 2018). Here, we sequenced the first chloroplast genome of *O. changjiangense*. Our aims were to: (1) characterize the structure, content of *O. changjiangense* chloroplast genome; (2) clarify the phylogenetic position of *O*. *changjiangense*.

Individuals of *O*. *changjiangense* were sampled from Sanya, Hainan, China (latitude 18.22°, longitude 109.60°) and stored in the greenhouse at Nanjing Normal University (http://www.njnu.edu.cn/, Zhitao Niu, niuzhitaonj@163.com) under the voucher number WMT20001. Fresh leaves were collected from *O*. *changjiangense* for DNA extraction. Total genomic DNA was isolated using DNeasy Plant Mini Kit (Qiagen, Germany). The DNA sample was deposited at Nanjing Normal University. The quality of the DNA sample was estimated by NanoDrop 8000 Spectrophotometer (Thermo Scientific, Wilmington, DE, USA) and agarose gel electrophoresis. DNA conformed to standard (concentration ≥50 ng/μl, A260/A280 = 1.8-2.0, A260/A230 > 1.8) were used to sequence at Illumina Hiseq4000 platform. Approximately 6 G raw data were generated and to obtain clean data, raw data were filtered by Fast QC. The clean data were trimmed and assembled by CLC Genomics Workbench 8.0 (CLC Bio, Aarhus, Denmark). Gaps and junctions between IR regions and SC regions were confirmed by PCR amplification. Genes were annotated by DOGMA (Wyman et al. 2004) and tRNAscan-SE 1.21 (Schattner et al. 2005). The complete chloroplast genome of *O*. *changjiangense* was submitted to DDBJ (Accession No. LC_579430).

The complete chloroplast genome of *O*. *changjiangense* was successfully assembled in this study. The total length of the chloroplast genome was 149,081bp. The chloroplast genome contained four regions including two IR regions (262,11 bp), an LSC region (828,69 bp), and an SSC region (137,90 bp). Total 110 genes were annotated including 76 protein-coding genes, 30 tRNA genes, and 4 rRNA genes. The total GC content of the chloroplast genome was 37.37% which was similar to other Orchidaceae species (Li et al. 2020). The GC content of different regions of the chloroplast genome ranged from 30.26% to 43.27%, and the IR region had the highest GC content.

To clarify the phylogenetic position of *O*. *changjiangense*, the maximum-likelihood (ML) tree of 17 Orchidaceae species was constructed based on complete chloroplast genomes (Figure 1). *Goodyera schlechtendaliana* Rchb. f. was selected as an outgroup. The ML tree was strongly supported (bootstrap supports > 95%). The results showed that *Oxystophyllum* was sister to *Phaius* with 100% support, which was consistent with previous studies (Niu et al. 2017). We considered that chloroplast genomes have strong power in phylogeny studies.

**Figure 1.**
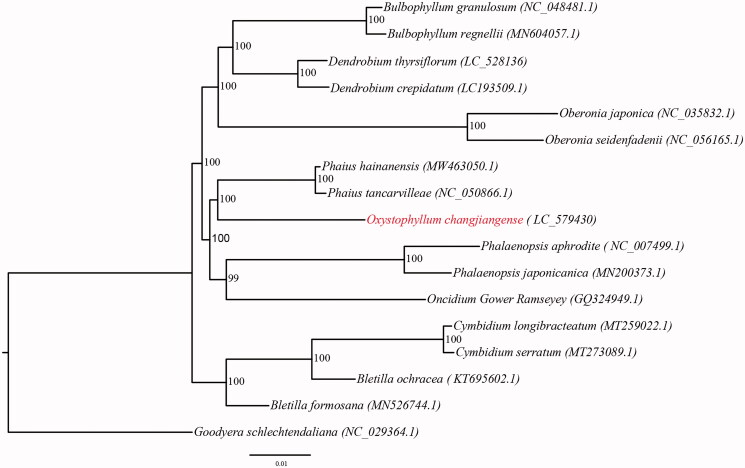
Maximum-likelihood tree of Orchidaceae species based on the whole chloroplast genome sequences with *Goodyera schlechtendaliana* as outgroup. Numbers near the nodes represent ML bootstrap values. *Oxystophyllum changjiangense* is highlighted in red. The contents in parentheses are accession numbers of chloroplast genomes. *Oxystophyllum changjiangense* is sister to *Phaius tancarvilleae* with 100% support.

## Data Availability

The genome sequence data that support the findings of this study are openly available in GenBank of NCBI at (https://www.ncbi.nlm.nih.gov/) under the accession number LC_579430. The associated **BioProject**, **SRA**, and **Bio-Sample** numbers are PRJNA669498, SRR12853059, and SAMN16442799 respectively.
